# Whole-Genome Resequencing Identifies *KIT* New Alleles That Affect Coat Color Phenotypes in Pigs

**DOI:** 10.3389/fgene.2019.00218

**Published:** 2019-03-18

**Authors:** Zhongping Wu, Zheng Deng, Min Huang, Yong Hou, Hui Zhang, Hao Chen, Jun Ren

**Affiliations:** State Key Laboratory of Pig Genetic Improvement and Production Technology, Jiangxi Agricultural University, Nanchang, China

**Keywords:** pig, coat color, *KIT*, genome resequencing, allele diversity

## Abstract

The Duroc × (Landrace × Large White) hybrid pig (DLY) is the most popular commercial pig used in the Chinese pig industry. DLY pigs are usually white but sometimes show colored phenotypes. Colored DLY pigs are not favored by slaughterhouses and retailers, thus causing certain economic losses to farmers in China. In this study, we first conducted a genome-wide association study and RNA sequencing to demonstrate that *KIT* variants are responsible for diversifying coat color phenotypes segregating in a DLY population. We then defined the precise sizes and locations of four duplications (DUP1-4), four candidate causative mutations at the *KIT* locus, in the pig reference genome using the whole-genome sequence data of representative colored individuals. The sequence data also enabled us to identify a list of new *KIT* alleles. By investigating the association between these new alleles and coat color phenotypes, we provide further evidence that DUP2 is another causative mutation for the solid white coat color in pigs. DUP1 (the *KIT* gene duplication), DUP2 and the splice mutation are all required for the manifestation of a solid white coat color. DUP4 had a more significant effect on the formation of the belt phenotype compared with DUP3. Given the necessity of DUP2 for the solid white coat color, we detected *I^N^*/*I^N^* homozygotes lacking DUP2 in Large White and Landrace pigs and found that French Landrace pigs had the highest frequency (8.98%) of *I^N^*/*I^N^* individuals. This study not only advances our understanding of the molecular mechanism of the color phenotype in pigs, but also establishes a simple and accurate method for the screening of *KIT I^N^*/*I^N^* homozygotes in Large White and Landrace that would cause colored DLY pigs.

## Introduction

Coat color is one of the most visualized breed features of domestic pigs. After long-term artificial selection, domestic pigs have formed diversifying coat color phenotypes (Porter, 1993; Legault et al., 1998), including black, red, white, spotted, brown, belted, two-end black, etc. The genetic studies of coat color in pigs trace back to the beginning of the last century (Spillman, 1906). To date, several major genes affecting pig coat color phenotypes have been identified. *MC1R* variants are responsible for the dominant black, black spotted, and red coat colors in both Western and Chinese pigs (Kijas et al., 1998, 2001; Fang et al., 2009). *KIT* variants determine the dominant white coat color in Western pigs (Johansson et al., 1996; Marklund et al., 1998). *MITF* mutation causes the recessive white coat color in Chinese Rongchang pigs (Chen et al., 2016). *ASIP* affects the black-and-tan coat color in Mangalitza pigs (Drögemüller et al., 2006). *TYRP1* and *EDNRB* variants are responsible for the brown (Ren et al., 2010) and white belt coat colors (Ai et al., 2013) in Chinese pigs, respectively.

Multiple alleles for different coat color phenotypes have been identified at the *KIT* locus, including the recessive wild-type allele *i*, *Patch* allele *I^p^* (Johansson et al., 1992, 1996), *Belt* allele *I^Be^* (Giuffra et al., 1999), *Roan* allele *I^Be^*^∗^ (Pielberg et al., 2002), and the *Dominant white* alleles *I* comprising *I*^1^, *I*^2^, *I*^3^ (Pielberg et al., 2002, 2003; Johansson et al., 2005). Recently, whole-genome resequencing has uncovered not only a 450 kb duplication (DUP1) encompassing the entire *KIT* gene, but also a 4.3-kb duplication (DUP2) ∼100 kb upstream of *KIT*, a 23-kb duplication (DUP3) ∼100 kb downstream of *KIT*, and another 4.3-kb duplication (DUP4) within DUP3 (Rubin et al., 2012). The wild-type *i* allele lacks the four duplications and the splice mutation, causing exon skipping in intron 17 (hereafter refer as to splice mutation). Allele *I^p^* carries DUP1 but not DUP2-4 and the splice mutation. Allele *I^Be^* carries DUP2-4 but lacks DUP1 and the splice mutation. The *I* alleles have variable copy numbers of DUP1-4 and the splice mutation. DUP1, the splice mutation and most likely DUP2 are known to be causative mutations for the dominant white coat color (Giuffra et al., 2002; Rubin et al., 2012). Unequal crossover is prone to occur in duplicated regions during homologous recombination (Zhang, 2003). It is thus likely to create great haplotype diversity due to the variable copy numbers of DUP1-4 at the *KIT* locus. However, new *KIT* haplotypes (alleles) in addition to the above-mentioned ones have not been reported yet, and the effect of these new alleles on pig coat color remains elusive.

**FIGURE 1:**
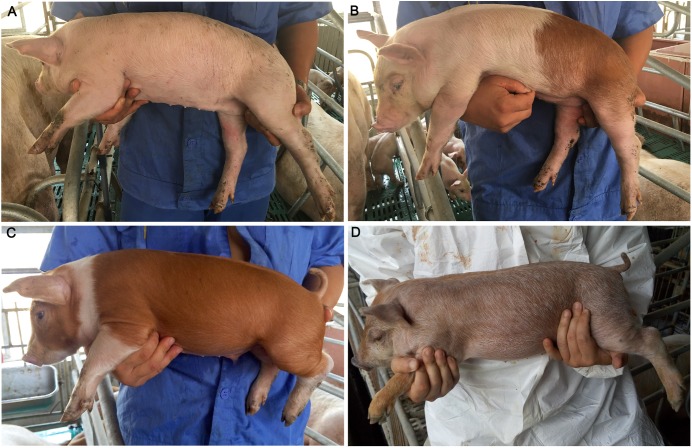
Coat color phenotypes in the tested population. **(A)** White. **(B)** White with reddish-brown spots. **(C)** Reddish-brown with white legs and belts. **(D)** Roan characterized by brown hairs intermingled with white hairs and most pigs had black spots on the snout.

Here, we first conducted a genome-wide association study (GWAS) and RNA sequencing, illustrating that *KIT* is a major locus for the coat color phenotypes (Figure 1) segregating in a commercial pig population derived from a three-way cross: Duroc × (Landrace × Large White) (hereafter refer as to DLY). Then, whole-genome sequence data (20× depth) of representative colored individuals from the DLY population identified new alleles (haplotypes) at the *KIT* locus. Finally, we established the relation of these *KIT* new alleles to pig coat color phenotypes. These findings advanced our understanding of the molecular mechanism of coat color phenotypes in domestic pigs, and also allowed us to establish a simple but robust PCR-based test for one new *KIT* allele causing colored individuals in DLY pigs.

## Materials and Methods

### Animals

In this study, ear tissue samples were collected from 190 DLY piglets raised in a commercial pig farm in Xinyu city, Jiangxi province, China. These piglets were derived from a three-way cross between American Duroc boars and French Landrace-Large White hybrid sows. Genomic DNA was extracted from the ear tissue of each pig using a genomic DNA extraction kit (Generay, China) according to the manufacturer’s instructions.

### Phenotypic Measurement

Four coat color phenotypes were observed in the 190 piglets, including (1) white, (2) white with reddish-brown spots, (3) reddish-brown with white legs and belts, (4) roan characterized by brown hairs intermingled with white hairs and most pigs had black spots on the snout (Figure 1). Each individual was recorded for its coat color phenotype using a cell phone camera. Graphic processing was performed to calculate the proportion of reddish-brown hair using PHOTOSHOP CS (Adobe Systems Incorporated, United States) as previously described (Fan et al., 2014). Due to bad photo angles, 87 individuals were discarded for the graphic processing and 103 individuals were used to calculate the proportion of reddish-brown hair. The calculated values were then classified into three scores: 0 for 0% (*n* = 20), 1 for 1–50% (*n* = 43), and 2 for 51–100% (*n* = 40). These scores were used as the coat color phenotypic values of the 103 individuals for subsequent analysis.

### Genome Wide Association Study

The 103 DLY piglets were genotyped for 68,528 SNPs using the Porcine 80K Genotyping array (Illumina, United States). SNPs were filtered using PLINK v1.90 (Purcell et al., 2007) by removing those with ambiguous locations, minor allele frequencies of less than 0.01, or genotyping rates of less than 0.95. Individuals with SNP call rates of less than 0.90 were also discarded. After the filtering process, 55,117 SNPs from the 103 individuals were retained for subsequent GWAS.

The GWAS was performed using a mixed linear model implemented in the Genome-wide Efficient Mixed-Model Association (GEMMA) software (Zhou and Stephens, 2012). The phenotypic values were regressed on sex and batch, and the residuals were used as the new phenotypes. Then, a univariate linear mixed model regression was executed with phenotypic trait, genotype, and relatedness matrix files (Wang et al., 2018). The threshold of genomic significance was set by the Bonferroni method: 0.05/the number of tested SNPs (Yang et al., 2005).

### RNA Sequencing

Four reddish-brown and four white dermal tissues were collected from DLY piglets raised in the same farm in Xinyu city, Jiangxi province, China. Dermal tissues were preserved in RNA later and stored at -80°C before use. Total RNA was extracted using the Trizol reagent (Invitrogen, United States) following the manufacturer’s protocol. RNA quality was assessed via agarose gel electrophoresis and a NanoDrop-1000 instrument (Thermo Fisher Scientific, United States).

Qualified RNA with an integrity number (RIN) greater than 6.8 were used for cDNA library construction using AmpliSeq for Illumina Library Plus (Illumina, United States). cDNA libraries were sequenced as paired-end 150 bp reads on an HiSeq 4000 instrument (Illumina, United States). RNA-seq reads were aligned against the pig reference genome (*Sscrofa*11.1) using STAR (Dobin et al., 2013). The counts of all annotated genes were calculated using STRingtie (Pertea et al., 2015) and featureCounts (Liao et al., 2014). Differentially expressed genes (DEGs) between white and reddish-brown skin samples were then determined using the DEseq2 software from Bioconductor (Love et al., 2014), under default parameters.

### *KIT* Genotyping

All DLY piglets were genotyped for the splice mutation via Sanger sequencing after PCR amplification using primers KIT_F (5′-CCCCGACTCTCCTAACAGTGTA-3′) and KIT_R (5′-TGCATGGTATGGCAAAGGTAG-3′). The DUP1 duplication breakpoint (DBP) was amplified using primers DUP1BP_F (5′-ATGTGGAGAAGCAGGAGACC-3′) and DUP1BP_R (5′-TGTTTCACCCGCATCCTACT-3′). PCR reaction was run on a Thermal Cycler (Bio-Rad, United States) at 95°C for 5 min, 30 × (95°C for 30 s, 60°C for 30 s, 72°C 40 s), 72°C 10 min and 12°C forever. PCR products were visualized through 1% agarose gel electrophoresis.

### Quantitative PCR and Droplet Digital PCR

Genomic quantitative (qPCR) were performed to detect the copy numbers of DUP1-4 using TaqMan primers and probes as previously descried (Rubin et al., 2012). PCR reactions were conducted using Probe qPCR Mix (TakaRa, Beijing) in a total volume of 10 μl containing 25 ng of genomic DNA, 0.2 μl of target and reference primers (10 μM), 0.1 μl of target and reference probe mix (10 μM), and 0.2 μl ROX Reference Dye. The genomic copy numbers of DUP1-4 were estimated in relation to the single copy locus *ESR1* using the ΔΔCt methodology (Livak and Schmittgen, 2001). The Ct value of each sample was obtained from a mean of three runs.

Droplet digital PCR (ddPCR) was explored to verify the genomic qPCR results as previously reported (Hindson et al., 2011; Pinheiro et al., 2012). In brief, genomic DNA was digested with the restriction enzyme *Spe*I-HF (NEB, United Kingdom) for 1 h at 37°C, then inactivated for 20 min at 80°C. ddPCR reaction mixture was prepared in a volume of 20 μl containing 1 ng digested DNA template, 10 μl 2 × ddPCR Supermix for Probes (Bio-Rad, United States), 1.8 μl each target or reference primer (10 μM, Supplementary Table S1) and 0.5 μl probe mix (10 μM, Supplementary Table S1). The reaction mixture was then emulsified using droplet generator oil (Bio-Rad, United States) and a QX200 Droplet Generator (Bio-Rad, United States). About 20,000 droplets were generated per sample. The droplets were then transferred to a 96-well and were amplified on a Thermal Cycler (Bio-Rad, United States) at 95°C for 10 min, 40 × (94°C for 30 s, 59°C for 1 min), 98°C for 10 min. After amplification, the droplets were read in the QX200 Droplet Reader (Bio-Rad, United States). The copy numbers of DUP1-4 in the tested samples were finally estimated using QuantaSoft version 1.7.4.0917 (Bio-Rad, United States). Each sample was run in duplicate, and unless otherwise specified, the copy numbers were the average of the two measurements.

### Whole-Genome Resequencing

Six colored DLY pigs including four individuals with the phenotypic score of 1 and two individuals with the phenotypic score of 2, four Landrace pigs and three Large White pigs were sequenced at 25-fold depth using a whole-genome shotgun strategy. Short-insert (350 bp) DNA libraries were paired-end sequenced on a HiSeq 2500 platform (Illumina, United States). The sequence data for each individual reached more than 25-fold depth. The paired-end short sequence reads were mapped to the *Sscrofa*11.1 reference genome using the BWA software (v.0.7.10) (Li and Durbin, 2009). Alignment files were then sorted and converted to BAM format via SAMtools software (v.1.6) (Li et al., 2009).

### Copy Number Variation Prediction

In addition to whole-genome sequence data of 13 individuals obtained in this study, two publicly available whole-genome sequence data sets were used for the prediction of copy number variations at the *KIT* locus. One data set included whole-genome sequences (∼10× depth) of 8 Duroc, 7 Pietrain, 4 Hampshire, 9 Landrace, and 17 Large White pigs (Accession numbers: ERP011076 and ERP001813) (Groenen et al., 2012; Frantz et al., 2015). The other one included whole-genome sequence (∼25× depth) data of 6 Chinese wild boars and 62 Chinese domestic pigs (Accession number: SRA096093) (Ai et al., 2015).

Copy number variation regions (CNVRs) on pig chromosome 8 were predicted for 13 re-sequenced individuals using CNVcaller (Wang et al., 2017) under default procedure and parameters in a 800 bp window size. A Gaussian hidden Markov model method was further explored (Miles et al., 2016) to predict the copy number state along the *KIT* locus for each individual alignment. In brief, we computed the coverage of aligned sequence reads across the DUP1 region encompassing the *KIT* gene and its 500 kb flanking region on each side (chr8: 40722801-42284000 bp) in 800 bp non-overlapping bins for each sample. Then, we excluded bins where the GC content was lower than 20% to eliminate coverage bias and normalized the coverage values to 2 by median depth for diploid copy number state analysis. At last, copy number state in the *KIT* region was predicted for all samples by fitting the Gaussian hidden Markov model to the normalized coverage data.

### PCR Amplification of Duplication Breakpoints

PCR primers (Supplementary Table S1) were designed to amplify DUP1-4 duplication breakpoint sequences. PCR reaction was performed in a volume of 25 μl containing 1.5 μl genomic DNA (50–100 ng/μl), 12.5 μl 2× Taq Master Mix (Dye) Plus (Vazyme, China), 0.5 μl forward primer (10 μM), 0.5 μl reverse primer (10 μM) (Sangon, China), and 10 μl water. PCR reaction was run on a Thermal Cycler (Bio-Rad, United States) at 95°C for 5 min, 30 × (95°C for 30 s, 57°C for 30 s, 72°C 1–2 min), 72°C 10 min and 12°C forever. After amplification, 1% agarose gel electrophoresis was performed to separate the amplified products with a voltage of 160 V and a time of 15 min. PCR products were directly sequenced to verify their identities.

## Results and Discussion

### GWAS Indicates That *KIT* Is the Major Gene Responsible for Coat Color Phenotypes in the DLY Population

**FIGURE 2:**
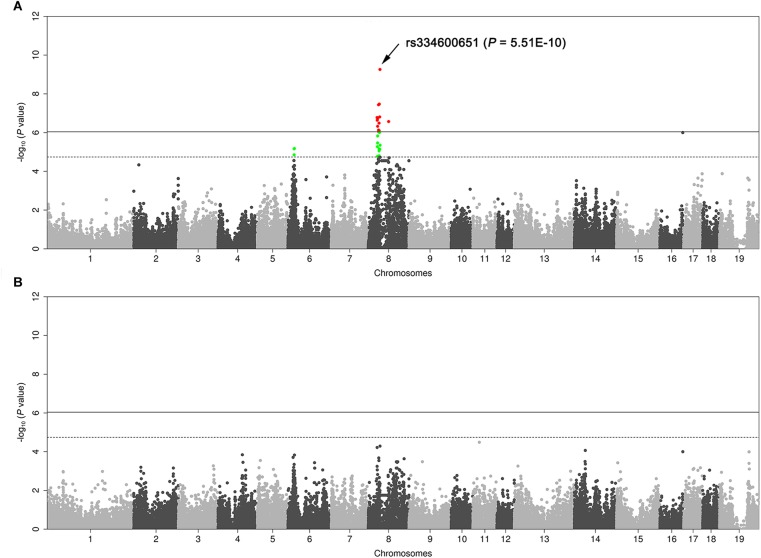
Manhattan plots of genome-wide association study (GWAS) for the proportion of reddish-brown hairs in 103 Duroc × (Landrace × Large White) piglets. **(A)** Routine GWAS. **(B)** Conditional GWAS. In the Manhattan plots, genomic positions of qualified SNPs are given in the *X*-axis, and the –log_10_ *P*-values for SNP associations with the phenotype are shown in the *Y*-axis. The red dots represent SNPs that exceed the 5% genome-wide significance threshold, and green dots represent SNPs surpassing the suggestive significance threshold. Solid and dashed lines indicate the 5% genome-wide and chromosome-wide (suggestive) Bonferroni-corrected thresholds, respectively.

We genotyped the 103 DLY pigs segregating for four coat color phenotypes using the Illumina 80K chip. GWAS identified a strong association signal for the proportion of reddish-brown hair on chromosome 8 (SSC8). A total of 16 SNPs on this chromosome surpassed the genome-wide significant threshold (*P* = 9.1E-7), and the strongest associated SNP was rs334600651 (*P* = 5.51E-10) at 40.78 Mb (*Sscrofa*11.1, Figure 2A), which was only 618 kb away from the *KIT* gene. We further determined the DUP1 genotypes (presence or absence) of the 103 DLY pigs by amplification of the DUP1 breakpoints with specific primers (Supplementary Table S1). We then conducted a conditional GWAS analysis in which the DUP1 genotypes were included as a fixed effect and did not observe any significant association signal across the genome afterward (Figure 2B). These results support that *KIT* variants are responsible for the coat color phenotypes segregating in the DLY population.

### RNA Sequencing Supports That *KIT* Is the Causative Gene for Coat Color Phenotypes in the DLY Population

**FIGURE 3:**
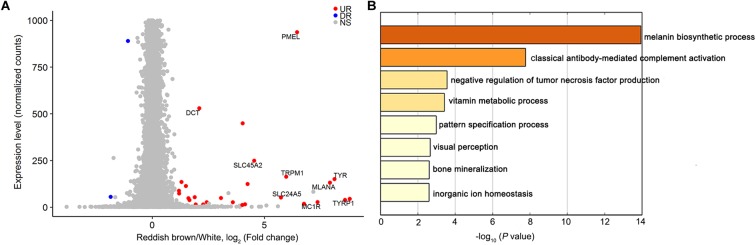
Differentially expressed genes between white and reddish-brown skin in DLY piglets. **(A)** Transcript levels (normalized gene counts) are plotted as a function of differential expression (log_2_-transformed fold change) between reddish-brown and white skin. Up-regulated expression genes in reddish-brown skin are shown in red. **(B)** Heatmap of enriched terms colored by *P*-value for differentially expressed genes between reddish-brown and white skin.

We used RNA sequencing to explore the genome-wide mRNA expression profile of the reddish-brown and white skins from DLY pigs. By comparing the transcriptome data of reddish-brown and white hair skin tissues, we identified 71 DEGs (*P* < 0.05, Figure 3A and Supplementary Table S2) that were over-represented in the *KIT*-mediated melanin biosynthetic process (Figure 3B). The expression of nine well-known pigmentation genes (*TYRP1*, *TYR*, *MC1R*, *PMEL*, *DCT*, *TRPM1*, *SLC24A5*, *MLANA*, and *SLC45A2*) were up-regulated in reddish brown skin [*P* < 0.05, Log_2_ (fold change)]. Given that DEGs between white and reddish-brown skin tissues were significantly enriched in the pigmentation biosynthetic pathway involving *KIT*, we assume that *KIT* is the causative gene for coat color phenotypes in the DLY population.

### *KIT* Genotypes Are Discordant With the Expected Coat Color Phenotypes in the DLY Population and Its Parental Lines

We genotyped the DUP1 and splice mutation of 190 DLY piglets using PCR and Sanger sequencing (see section “Materials and Methods”). At the splicing mutation site, 143 individuals had *AG* genotypes and 47 were *GG* individuals (Supplementary Table S3). Of the 143 individuals, 140 were identified as *I*/*i* as they carried DUP1 and the *AG* genotype at the splice mutation site. Unexpectedly, only 64 out of the 143 individuals showed solid white coat color, and the others were colored individuals, which was inconsistent with the expectation that all individuals should be white. Moreover, three DLY piglets lacked DUP1 and showed the *AG* genotype at the splice mutation site, indicating that these individuals carried the homozygous lethal *Dominant white* allele *I^L^* (Pielberg et al., 2003). The 47 individuals should be *i*/*i* at the *KIT* locus as they lacked DUP1 and had the *GG* genotype at the splice mutation site. Of the 47 individuals, 18 had a reddish-brown coat color with white belts and 29 showed roan hairs (Supplementary Table S3), suggesting that two different new *i* alleles are presented in these individuals.

According to Rubin et al. (2012), the ratios of DUP2/DUP1 and DUP3/DUP1 in white (*I*/-) individuals are 1.5–3. We selected 12 DLY individuals representing three coat color phenotypes, i.e., reddish-brown with white belts, reddish-brown with white legs and roan (Figure 1B–D) and determined the splice mutation genotype via Sanger sequencing and the genomic copy numbers of DUP1-4 for each individual using qPCR. We found that both the ratios of DUP2/DUP1 and DUP3/DUP1 were less than 1.5 in the *I*/*i* individuals with a reddish-brown coat color with white legs and belts. However, both the ratios of DUP2/DUP1 and DUP3/DUP1 were greater than 1.5 in the *i*/*i* individuals with a roan coat color (Supplementary Table S4).

We further selected 10 Landrace and five Large White pigs from the parental farms of these DLY individuals and also detected their copy numbers of DUP1-4 using qPCR. The ratios of DUP2/DUP1 and DUP3/DUP1 were less than 1.5 in four Landrace pigs, and the ratios of DUP3/DUP1 were less than 1.5 in three Landrace and two Large White (Supplementary Table S4). Based on the copy numbers of DUP1-4, we deduced the *KIT* haplotypes of these tested Large White, Landrace and DLY individuals. The *I* alleles most likely lacked DUP2-3 in the *I*/*i* DLY individuals with the ratios of DUP2/DUP1 and DUP3/DUP1 less than 1.5. These previously unreported *I* alleles (denoted as *I^N^*) could be formed by an unequal crossover at the *KIT* locus during homologous recombination. For the *i*/*i* DLY individuals with the ratios of DUP2/DUP1 and DUP3/DUP1 more than 1.5, these individuals probably carried new *i* allele (*i^N^*) that have multiple copies of DUP2/3 but lack DUP1. Additional *KIT* new alleles likely exist in Landrace and Large White individuals with the ratios of DUP2/DUP1 and DUP3/DUP1 less than 1.5 (Supplementary Table S4).

### Whole-Genome Resequencing Identifies Previously Unreported Alleles of *KIT*

To verify our speculation of the *KIT* new alleles, we conducted whole-genome resequencing at 20× depth on 13 pigs including six colored DLY, four Landrace and two Large White pigs that were predicted to carry *KIT* new alleles as mentioned above and one normal Large White pig. First, to determine the precise position of DUP1 in the pig reference genome (*Sscrofa*11.1), we used CNVcaller (Wang et al., 2017) to predict the copy number variation regions (CNVRs) of the 13 re-sequenced individuals on chromosome 8. After merging the CNVRs of all individuals, we observed a 561-kb shared CNVR (chr8: 41222801-41784000 bp) encompassing the entire *KIT* gene (Supplementary Table S5). The CNVR perfectly correspond to the duplicated *KIT* (DUP1) region (hereafter refer as to the 561-kb region), and its size is larger than the previously reported one (450 kb) (Rubin et al., 2012).

**FIGURE 4:**
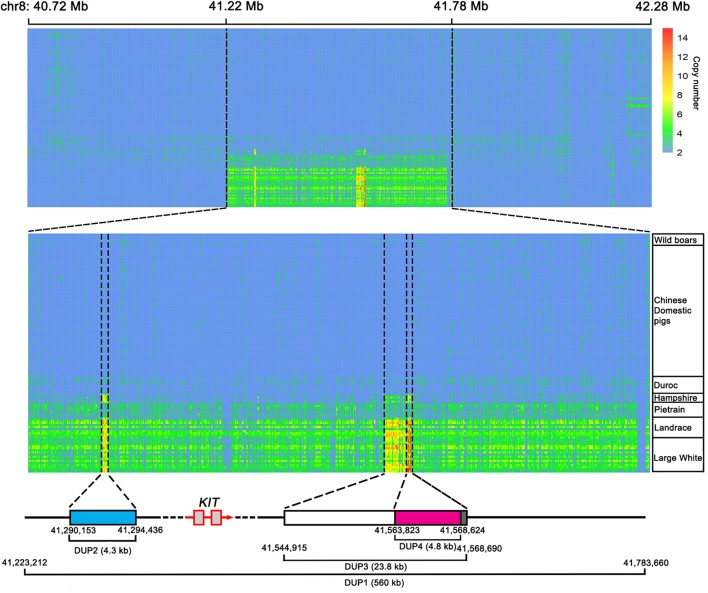
Heatmap of copy number prediction along the *KIT* locus for 113 individually sequenced pigs retrieved from the NCBI database. For each individual, diploid copy numbers were predicted in 800 bp non-overlapping bins by normalized coverage of DUP1 and its 500 kb flanking region on each side. The copy numbers of DUP1 are shown in magnifications to the middle. The accurate starting and ending positions of DUP1-4 were confirmed by Sanger sequencing.

Next, we predicted the copy number variation (CNV) within the 561-kb region using the hidden Markov model (HMM) method described by Miles et al. (2016). To test the reliability of the HMM method, we downloaded the whole-genome sequence data of 113 individuals representing Chinese wild boars, Chinese domestic pigs and Western modern breeds (Duroc, Hampshire, Pietrain, Landrace, and Large White) from the NCBI public database. The diploid copy numbers were predicted in an 800 bp window for the 561-kb region and its 500-kb flanking region (chr8: 40722801-42284000 bp). We found that all Chinese wild boars and domestic pigs and Western Duroc pigs did not have CNV within this region. Pietrain had 3–4 copies of DUP1, Landrace and Large White had 3–6 copies of DUP1, and Hampshire carried DUP2-4 but not DUP1 (Figure 4). This observation was consistent with the previous report by Rubin et al. (2012) and thus supports the reliability of HMM method for CNV prediction.

Further, we designed specific primers to amplify duplication breakpoints of DUP1-4 based on the CNV prediction results in the 561-kb region. PCR amplification was performed using the genomic DNA of Large White pigs having DUP1-4. Sanger sequencing defined the accurate start and stop positions of DUP1-4 in the *Sscrofa*11.1 genome assembly into chr8: 41223212-41783660 bp (560 kb), chr8: 41290153-41294436 bp (4.3 kb), chr8: 41544915-41568690 bp (23.8 kb), and chr8: 41563823-41568624 bp (4.8 kb), respectively (Figure 4).

**FIGURE 5:**
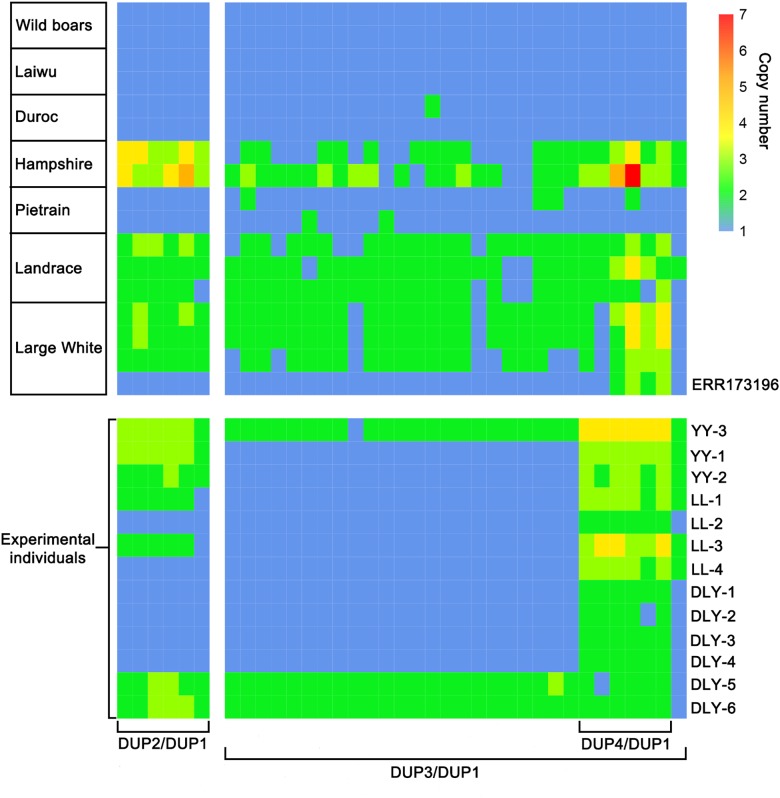
Heatmap of the ratios of DUP2-4 to DUP1 in 17 representative individuals retrieved from the NCBI database and 13 individuals re-sequenced in this study. ERR173196, Accession number in the NCBI database; LL, Landrace; YY, Large White; DLY, Duroc × (Landrace × Large White).

Then, we applied the HMM method to predict the diploid copy number variation in the 560-kb region for the 13 re-sequenced individuals. By comparing the CNV prediction results of these individuals with those of the 113 individuals from NCBI public database, we found abnormal copy numbers of DUP2 and DUP3 in one Large White pig from public database and 12 re-sequenced individuals, which could carry unreported *KIT* new alleles (Supplementary Figure S1). To clarify the *KIT* haplotypes of these individuals, we calculated the copy number ratios of DUP2-4 to DUP1 for each individual, confirming that one Large white pig from the public database and 12 re-sequenced individuals carried unreported *KIT* haplotypes. The ratios of DUP2/DUP1 and DUP3/DUP1 were 1.0 and the ratios of DUP4/DUP1 were 2.0–3.0 in four DLY individuals, two Landrace pigs and one Large White pig from public database. Two Large White and two Landrace pigs had 2.0–3.0 ratios of DUP2/DUP1 and DUP4/DUP1 and 1.0 ratio of DUP3/DUP1. The ratios of DUP2/DUP1 were 3.0 and the ratios of DUP3/DUP1 and DUP4/DUP1 were 2.0 in two DLY individuals with the *i*/*i* genotype (DLY-5 and DLY-6) (Figure 5).

**FIGURE 6:**
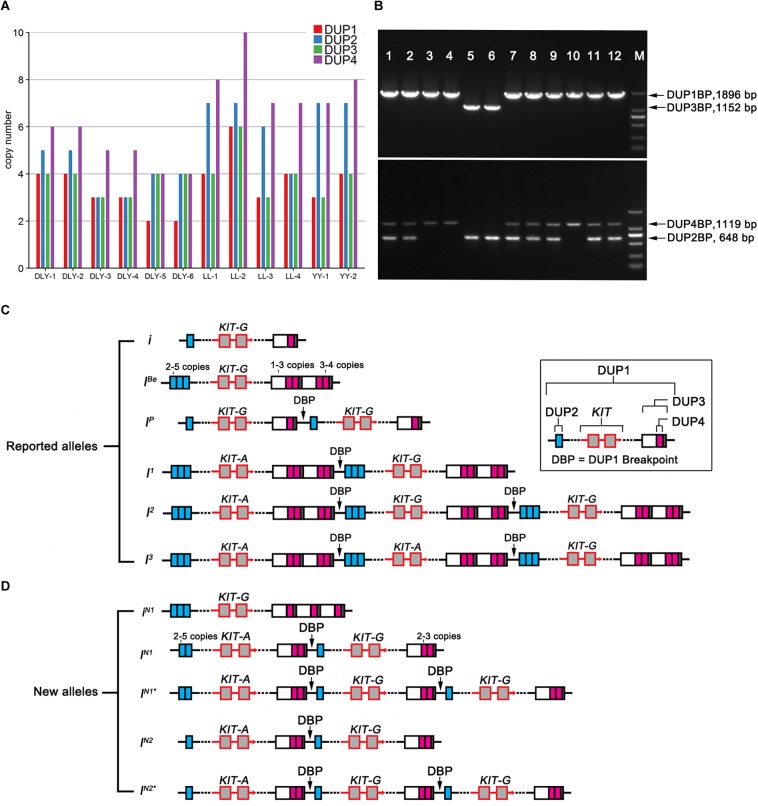
Copy number variations and deduced alleles at the *KIT* locus. **(A)** Genomic copy numbers of DUP1-4 in 12 re-sequenced individuals determined by ddPCR. **(B)** Amplification results of duplication breakpoint sequences DUP1 (DUP1BP, 1896 bp), DUP2 (DUP2BP, 648 bp), DUP3 (DUP3BP, 1152 bp), and DUP4 (DUP4BP, 1119 bp) in 12 pigs, Marker 2000 bp. **(C)** Schematic presentation of the previously reported *KIT* alleles adopted from Rubin et al. (2012) with slight modifications. **(D)** Schematic presentation of new *KIT* alleles identified in 12 re-sequenced individuals.

To accurately decipher the allelic structure of the re-sequenced individuals at the *KIT* locus, we determined the copy numbers of DUP1-4 (Figure 6A) and the ratios of G to A at the splice mutation (data not shown) in these individuals using Droplet digital PCR (ddPCR) (see section “Materials and Methods”) and amplified DUP1-4 breakpoint sequences (Figure 6B). In comparison with the *KIT* allelic structure reported by Rubin et al. (2012; Figure 6C), we identified five *KIT* new alleles: *i*^*N*1^ with multiple copies of DUP2/3 but lacking DUP1/4 and the splice mutation, *i*^*N*1^ and *I*^*N*1∗^ carrying multiple copies of DUP1, DUP2, DUP4 and the splice mutation but not DUP3, *I*^*N*2^ and *I*^*N*2∗^ with multiple copies of DUP1, DUP4 and the splice mutation but without DUP2 and DUP3 (Figure 6D).

### The Effect of *KIT* New Alleles on Pig Coat Color Phenotypes

**FIGURE 7:**
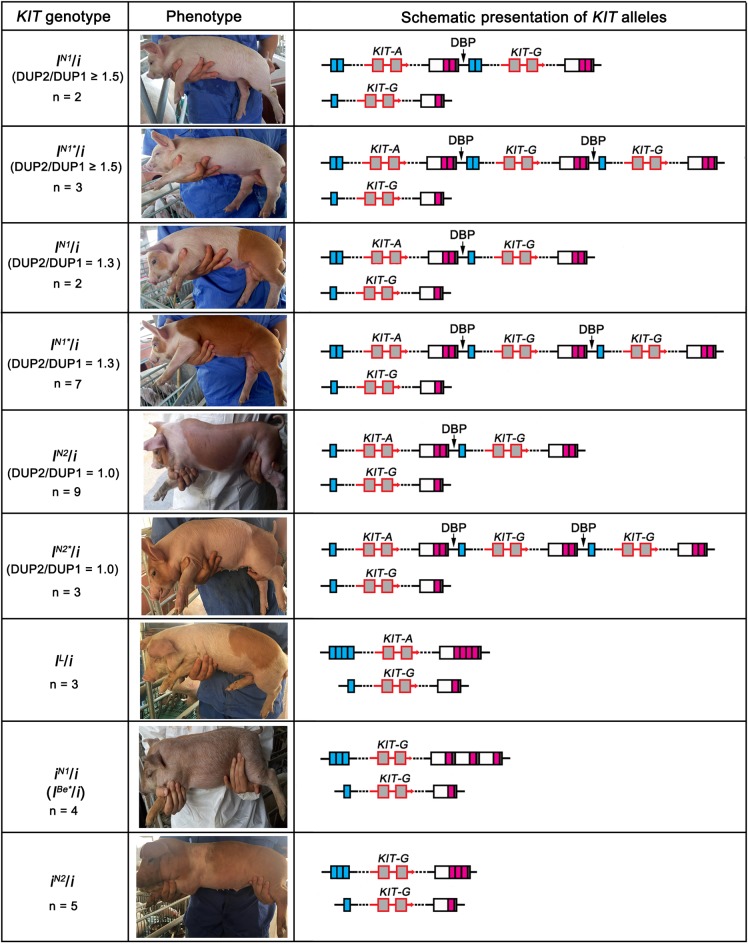
Association of *KIT* new alleles with coat color phenotypes in DLY pigs.

To investigate the effect of *KIT* new alleles on coat color phenotype, we determined the *KIT* genotypes of the 190 DLY pigs via amplification of DUP1-4 breakpoint sequences and the splice mutation (Table 1). In addition to 58 *I*/*i* individuals carrying the normal *KIT* Dominant White alleles *I* (including *I*^1^, *I*^2^, or *I*^3^), we identified 36 *i*^*N*1^/*i* or *I^N∗^*/*i* individuals lacking DUP3, 46 *I*^*N*2^/*i* or *I*^*N*2∗^/*i* animals lacking both DUP2 and DUP3, three *I^L^*/*i* pigs having the splice mutation but not DUP1, 29 *i*^*N*1^/*i* and 18 *I*^*N*2^/*i* pigs. The *I*^*N*2^ allele differs from *i*^*N*1^ in that it had multiple copies of DUP2 and DUP4 but not DUP1, DUP3 and the splice mutation (Figure 7).

**Table 1:** Deduced *KIT* genotypes in 190 DLY pigs.

*KIT*		Splice	
genotype	No.	mutation	DUP1BP	DUP2BP	DUP3BP	DUP4BP
*I*/*i*	58	*AG*	Yes	Yes	Yes	Yes
*i*^*N*1^/*i* or *I*^*N*1∗^/*i*	36	*AG*	Yes	Yes	No	Yes
*I*^*N*2^/*i* or *I*^*N*2∗^/*i*	46	*AG*	Yes	No	No	Yes
*I^L^*/*i*	3	*AG*	No	Yes	No	Yes
*i*^*N*1^/*i*	29	*GG*	No	Yes	Yes	No
*I*^*N*2^/*i*	18	*GG*	No	Yes	No	Yes


*KIT genotypes of these individuals were deduced based on the presence (yes) or absence (no) of DUP1-4 breakpoint sequences and the genotype of the splice mutation in intron 17 in the KIT gene. DUP1BP, DUP1 breakpoint; DUP2BP, DUP2 breakpoint; DUP3BP, DUP3 breakpoint; DUP4BP, DUP4 breakpoint. I^*N1*∗^ and I^*N2*∗^ may differ from I^*N1*^ and I^*N2*^ in copy numbers of DUP1-4.*

We then analyzed the association between the *KIT* genotypes and the coat color phenotypes of these 190 individuals. As expected, the 58 *I*/*i* DLY individuals had a solid white coat color. We randomly selected five individuals to perform ddPCR for quantifying the copy numbers of DUP1-4, and found that the ratios of DUP2/DUP1 and DUP3/DUP1 were 1.7–2.0 in all *I*/*i* individuals (Figure 7 and Supplementary Table S6), which is consistent with the previous report (Rubin et al., 2012).

We observed three coat color phenotypes in the 36 *i*^*N*1^/*i* or *I*^*N*1∗^/*i* individuals: white, white with reddish-brown spots and reddish-brown with white legs and belts. The ratios of DUP3/DUP1 were 1.0 and the ratios of DUP4/DUP1 were greater than 1.5 in all 36 individuals as revealed by ddPCR. However, the ratio of DUP2/DUP1 varied in these individuals. All individuals (*n* = 5) with a DUP2/DUP1 ratio of no less than 1.5 had the solid white coat color. When individuals had a DUP2/DUP1 ratio of 1.3 (i.e., most likely having one DUP2, *n* = 9), they displayed colored phenotypes even these animals carried DUP1, DUP4 and the splice mutation (Figure 7 and Supplementary Table S6). This indicates that more than one DUP2 is required for the formation of the solid white coat color in pigs.

For the 46 *I*^*N*2^/*i* or *I*^*N*2∗^/*i* individuals, all of them showed colored phenotypes including white with reddish-brown spots and reddish-brown with white legs and belts. The ddPCR analysis showed that the ratios of DUP2/DUP1 and DUP3/DUP1 were 1.0 and the ratios of DUP4/DUP1 were greater than 1.5 in these individuals (Figure 7 and Supplementary Table S6). This again highlights the importance of DUP2 in determining the solid white coat color in pigs and supports the previous assumption that DUP2 is another causative mutation for the Dominant White coat color in addition to DUP1 (the *KIT* gene duplication) and the splice mutation (Rubin et al., 2012).

We noticed that three DLY individuals carried the homozygous lethal allele *I^L^* (Figure 7 and Supplementary Table S6), which lacked DUP1 and DUP3 but had DUP2, DUP4 and the splice mutation. The coat color phenotypes of these three individuals were all white with reddish-brown spots (Figure 7 and Supplementary Table S6), suggesting that the splice mutation, DUP2 and DUP4 can collectively cause white coat color even without the *KIT* gene duplication (DUP1). When we looked at the 29 *i*^*N*1^/*i* individuals, all of which had the roan phenotype (Figure 7 and Supplementary Table S6). Allele *i*^*N*1^ carries DUP2 and DUP3 but not DUP1, DUP4 and the splice mutation, which is similar to the *I^Be^* allele carrying DUP2-4 but not DUP1 and the splice mutation, and is most likely the previously reported *I^Be∗^* allele causing the roan coat color in *I^Be∗^*/*i* individuals (Pielberg et al., 2002). This finding indicates that DUP4 is required for manifestation of the Belt phenotype of the *I^Be^* allele.

We then focused on the 18 *I*^*N*2^/*i* individuals that carried DUP2 and DUP4 but not DUP1, DUP3 and the splice mutation. These individuals all showed a reddish-brown coat color with white belts (Figure 7 and Supplementary Table S6), suggesting that DUP2 and DUP4 could result in a belt coat color in the absence of DUP1 and the splice mutation. Rubin et al. (2012) measured the copy numbers of DUP1-4 in four belted breeds including Angler Sattelschwein, British Saddleback, Cinta Senese, and Hampshire pigs. They found that all individuals had DUP2 and DUP4 but lacked DUP1, and a few individuals did not have DUP3, which allowed them to assume that both DUP2 and DUP4 contribute to the belt coat color phenotype. Our findings are consistent with their assumption. We noticed that both alleles *i*^*N*1^ and *I*^*N*2^ lack DUP1 and the splice mutation. The difference between the two alleles was that the *i*^*N*1^ allele has DUP2 and DUP3 but not DUP4, and is responsible for the roan phenotype, while the *I*^*N*2^ allele carries DUP2 and DUP4 but not DUP3 and leads to the reddish-brown coat color with white belts. This clearly suggests that DUP4 had a more significant effect on the formation of the belt phenotype compared with DUP3.

To validate the effect of the *KIT* new alleles on coat color phenotype, we selected two *I*^*N*2^/*I^L^* Landrace sows to cross with Duroc boars (*i*/*i*), generating a total of 23 Duroc × Landrace (DL) hybrid offspring. We further determined the *KIT* genotypes of the 23 DL individuals using ddPCR of DUP1-4, amplification of DUP1-4 breakpoints and Sanger sequencing of the splice mutation. Of the 23 individuals, four had the *I^L^*/*i* genotype and white coat color with reddish-brown spots. Nineteen DL individuals were colored pigs with a DUP3/DUP1 ratio of 1.0 and DUP2/DUP1, DUP4/DUP1 ratios of 1.3 (Supplementary Table S7). These observation were consistent with our finding of the coat color phenotypes in *I^L^*/*i* and *I*^*N*2^/*i* DLY pigs.

### Homozygotes of *I^N^*/*I^N^* Lacking DUP2 and Causing Colored DLY Pigs Are Presented in French Landrace Pigs at a Higher Frequency

**FIGURE 8:**
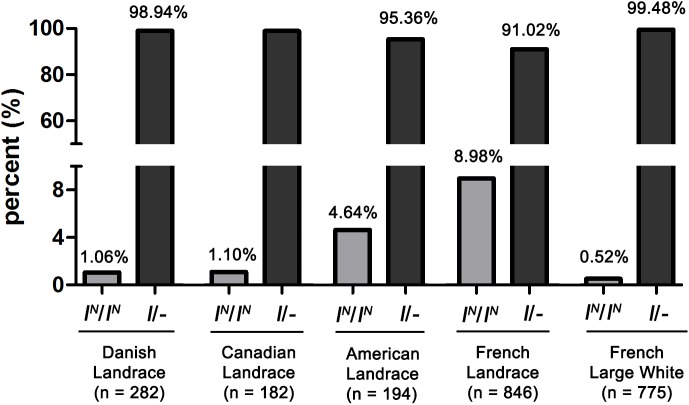
The frequency of *I^N^*(*I*^*N*2^ or *I*^*N*2∗^)/*I^N^* homozygotes lacking DUP2 in Landrace and Large White pigs.

Considering that more than one DUP2 is essential for the manifestation of the solid white coat color, and *I^N^* (*I*^*N*2^ or *I*^*N*2∗^)/*i* DLY pigs lacking DUP2 always show colored phenotypes, we detected the proportion of *I^N^*/*I^N^* homozygotes in 1504 Landrace and 775 Large White pigs from different countries using PCR amplification of DUP2 breakpoint sequences. We found that the frequency of *I^N^*/*I^N^* individuals was generally higher in Landrace than in Large White pigs, and French Landrace pigs had the highest frequency (8.98%) of *I^N^*/*I^N^* individuals (Figure 8). In this study, we sampled DLY pigs that were derived from Duroc boars × (French Landrace boars × French Large White sows) in a commercial pig farm. This explains why a large number of colored DLY individuals were observed in the farm where we collected samples. The PCR-based test for DUP2 established in this study can be explored to efficiently detect the *I^N^*/*I^N^* homozygotes in Landrace and Large White pigs, which would be helpful in reducing the number of colored DLY pigs and consequently prevent the economic loss for commercial farms in China where solid white DLY commercial pigs are favored.

## Conclusion

*KIT* variants are responsible for the coat color phenotypes segregating in the DLY population. DUP1, DUP2 and the splice mutation are all required for manifestation of a solid white coat color phenotype. In DLY pigs, DUP2 and the splice mutation can cause a white coat color with reddish-brown spots even in the absence of DUP1. DUP2 and DUP4 can result in a belt coat color phenotype in DLY pigs even without DUP1 and the splice mutation. Moreover, the solid white coat color cannot be formed in DLY pigs lacking DUP2 even if they carry DUP1 and the splice mutation. The PCR-based test for DUP2 provides a robust and simple tool for detecting *I^N^*(*I*^*N*2^ or *I*^*N*2∗^)/*I^N^* homozygotes in Large White and Landrace pigs. These homozygotes cause colored DLY pigs and segregate in French Landrace pigs at the highest frequency in comparison to other Large White and Landrace populations.

## Ethics Statement

All procedures used for this study and involving animals were in compliance with guidelines for the care and utility of experimental animals established by the Ministry of Agriculture of China.

## Author Contributions

JR designed the study and analyzed the data. JR and ZW wrote the manuscript. ZW, MH, and HC performed the bioinformatic analyses. ZW, ZD, YH, and HZ collected the data and performed the sequencing and genotyping experiments.

## Conflict of Interest Statement

The authors declare that the research was conducted in the absence of any commercial or financial relationships that could be construed as a potential conflict of interest.

## Supplementary Material

Figure S1 Heatmap of copy number prediction in the *KIT* region. For each individual, diploid copy numbers were predicted in 800 bp non-overlapping bins by normalized coverage of DUP1 and its 500 kb flanking region on each side. The heatmap of DUP2-4 and the *KIT* gene of the representative individuals retrieved from the NCBI database and 13 re-sequenced individuals are shown in the top and bottom, respectively. ERR173196, Accession number in the NCBI database; LL, Landrace; YY, Large White; DLY, Duroc × (Landrace × Large White).

Table S1 Primers and probes for detecting genomic copy numbers and amplifying duplication breakpoint sequences at the *KIT* locus. ^∗^5′ labeled with HEX and 3′ labeled with BHQ1. The other probes were 5′ labeled with 6-FAM and 3′ labeled with BHQ1.

Table S2 Differentially expressed genes between reddish brown and white skin of DLY piglets.

Table S3 *KIT* genotypes and coat color phenotypes in the tested DLY population.

Table S4 Genomic copy numbers of DUP1-4 in 27 pigs estimated by qPCR. DLY, Duroc × (Landrace × Large White); LL, Landrace; YY, Large White.

Table S5 Copy number variations in the 561-kb region containing the *KIT* gene in 13 re-sequenced individuals. DLY, Duroc × (Landrace × Large White); LL, Landrace; YY, Large White.

Table S6 Association of *KIT* genotypes and alleles with coat color phenotypes. Genomic copy numbers of DUP1-4 of these individuals were measured by ddPCR.

Table S7 *KIT* genotypes and coat color phenotypes in 23 Duroc × Landrace hybrid piglets.
