# Supplementary figures and images for: Whole-Genome Resequencing Identifies KIT New Alleles That Affect Coat Color Phenotypes in Pigs
Source: Front Genet. 2019 Mar 18;10:218. doi: 10.3389/fgene.2019.00218 (PMC6436083; doi:10.3389/fgene.2019.00218)

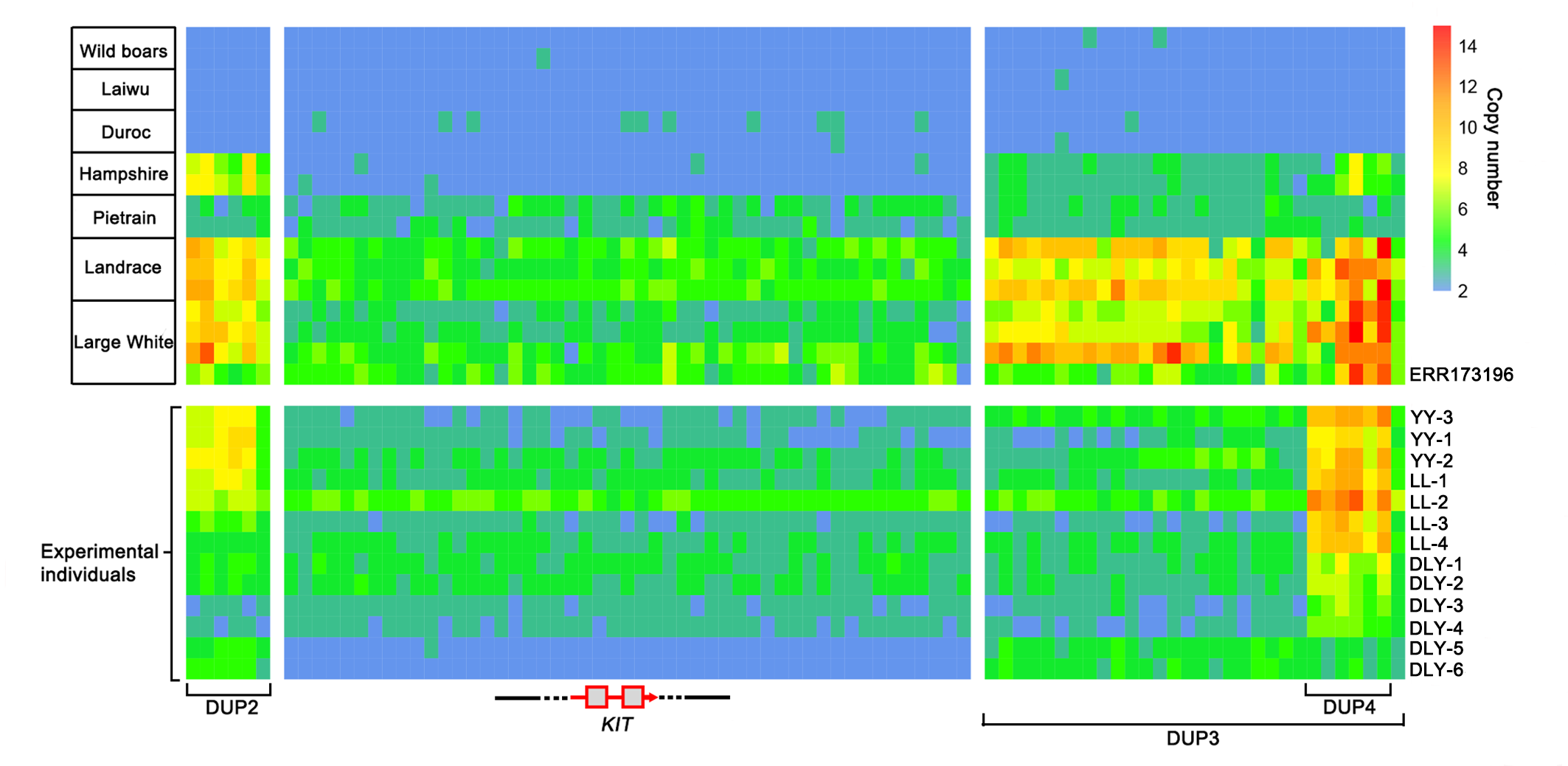

Supplement: Figure S1 — Heatmap of copy number prediction in the KIT region. For each individual, diploid copy numbers were predicted in 800 bp non-overlapping bins by normalized coverage of DUP1 and its 500 kb flanking region on each side. The heatmap of DUP2-4 and the KIT gene of the representative individuals retrieved from the NCBI database and 13 re-sequenced individuals are shown in the top and bottom, respectively. ERR173196, Accession number in the NCBI database; LL, Landrace; YY, Large White; DLY, Duroc × (Landrace × Large White). [file Image_1.TIF]
