# Supplementary material for: Whole-Genome Resequencing Identifies KIT New Alleles That Affect Coat Color Phenotypes in Pigs
Source: Front Genet. 2019 Mar 18;10:218. doi: 10.3389/fgene.2019.00218 (PMC6436083; doi:10.3389/fgene.2019.00218)
Supplement: Table S1 — Primers and probes for detecting genomic copy numbers and amplifying duplication breakpoint sequences at the KIT locus. ∗5′ labeled with HEX and 3′ labeled with BHQ1. The other probes were 5′ labeled with 6-FAM and 3′ labeled with BHQ1. [file Table_1.docx]

**Table S1** Primers and probes for detecting genomic copy numbers and amplifying duplication breakpoint sequences at the *KIT* locus

| Name | Type | Target | Sequence (5’ -3’) |
| --- | --- | --- | --- |
| CNV detection | | | |
| DUP1_F | Forward primer | DUP1 | GGATGCTACATGCAGCTGTT |
| DUP1_R | Reverse primer | DUP1 | GTTTGATGATGTGGGCTGAG |
| DUP1_P | Probe | DUP1 | TCAAGCTCACCACCTGGCATTC |
| DUP2_F | Forward primer | DUP2 | TGTGACCAGGAAGGGTAGTG |
| DUP2_R | Reverse primer | DUP2 | CAGGTGGGGCTTCTCTCTAT |
| DUP2_P | Probe | DUP2 | CCTCACTGCTCCTGCTAATTCCTC |
| DUP3_F | Forward primer | DUP3 | GGACGCATAAATTGTATGTAGATTG |
| DUP3_R | Reverse primer | DUP3 | TTCATTTTTGCACATTCCTTCT |
| DUP3_P | Probe | DUP3 | TGCCTGCTAACAATTATGCATGCA |
| DUP4_F | Forward primer | DUP4 | GCTCAGGGACCACAGGTATT |
| DUP4_R | Reverse primer | DUP4 | TATTTGGCAGGTGTTGGAAA |
| DUP4_P | Probe | DUP4 | AGCCTTCTAGCAGCTGATTGGATGG |
| ER_F | Forward primer | Estrogen receptor | ACGGTGGATATGGTCCTTCT |
| ER_R | Reverse primer | Estrogen receptor | CGCTCTCTCTCTGCACATTC |
| ER_P* | Probe | Estrogen receptor | TCAGGGTGCTGGACAGAAACG |
| Duplication breakpoint amplification | | | |
| DUP1BP_F | Forward primer | DUP1 Breakpoint | ATGTGGAGAAGCAGGAGACC |
| DUP1BP_R | Reverse primer | DUP1 Breakpoint | TGTTTCACCCGCATCCTACT |
| DUP2BP_F | Forward primer | DUP2 Breakpoint | GACAAGTGTGAAAACAGAAGTGTTA |
| DUP2BP_R | Reverse primer | DUP2 Breakpoint | TGTTTACATGCCAGGGGTTCAG |
| DUP3BP_F | Forward primer | DUP3 Breakpoint | AGGCTTGGCACTCAGATGAC |
| DUP3BP_R | Reverse primer | DUP3 Breakpoint | TCAGCCTCCCATCACAGGTA |
| DUP4BP_F | Forward primer | DUP4 Breakpoint | CAGTGGTGATGGAGGCTTGG |
| DUP4BP_R | Reverse primer | DUP4 Breakpoint | GGGGTTAGGCCTTAAGATGGT |

*5’ labeled with HEX and 3’ labeled with BHQ1. The other probes were 5’ labeled with 6-FAM and 3’ labeled with BHQ1.

**Table S3** *KIT* genotypes and coat color phenotypes in the tested DLY population

| *KIT* genotype | No. | Splice mutation | DUP1 breakpoint | White | White with reddish brown spots | Reddish brown with white legs and belts | Roan |
| --- | --- | --- | --- | --- | --- | --- | --- |
| *I*/*i* | 140 | *AG* | Presence | 64 | 25 | 51 | 0 |
| *I^L^*/*i* | 3 | *AG* | Absence | 0 | 3 | 0 | 0 |
| *i*/*i* | 47 | *GG* | Absence | 0 | 0 | 18 | 29 |

**Table S4** Genomic copy numbers of DUP1-4 in 27 pigs estimated by qPCR

| Pig | Phenotype | Splice mutation | DUP1 | DUP2 | DUP3 | DUP4 | DUP2/DUP1 | DUP3/DUP1 | DUP4/DUP1 | *KIT* genotype |
| --- | --- | --- | --- | --- | --- | --- | --- | --- | --- | --- |
| DLY-1 | Reddish brown with white belts | *AG* | 3.5 | 4.1 | 4.2 | 9.0 | 1.2 | 1.1 | 2.6 | *I^N^*/*i* |
| DLY-2 | Reddish brown with white belts | *AG* | 3.6 | 3.9 | 4.5 | 8.9 | 1.1 | 1.3 | 2.5 | *I^N^*/*i* |
| DLY-3 | Reddish brown with white legs | *AG* | 3.3 | 3.6 | 3.6 | 7.1 | 1.1 | 1.1 | 2.2 | *I^N^*/*i* |
| DLY-4 | Reddish brown with white legs | *AG* | 3.1 | 3.6 | 3.3 | 7.6 | 1.2 | 1.1 | 2.5 | *I^N^*/*i* |
| DLY-5 | Roan | *GG* | 1.8 | 2.8 | 4.2 | 4.0 | 1.6 | 2.4 | 2.2 | *i^N^*/*i* |
| DLY-6 | Roan | *GG* | 1.7 | 2.9 | 4.0 | 4.0 | 1.7 | 2.3 | 2.4 | *i^N^*/*i* |
| DLY-7 | Reddish brown with white belts | *AG* | 2.8 | 3.8 | 3.2 | 7.4 | 1.4 | 1.1 | 2.6 | *I^N^*/*i* |
| DLY-8 | Reddish brown with white belts | *AG* | 3.6 | 4.6 | 4.1 | 8.5 | 1.3 | 1.1 | 2.4 | *I^N^*/*i* |
| DLY-9 | Reddish brown with white legs | *AG* | 3.2 | 3.1 | 3.5 | 6.8 | 1.0 | 1.1 | 2.1 | *I^N^*/*i* |
| DLY-10 | Reddish brown with white legs | *AG* | 2.9 | 3.1 | 3.2 | 7.5 | 1.1 | 1.1 | 2.6 | *I^N^*/*i* |
| DLY-11 | Roan | *GG* | 1.7 | 3.1 | 3.2 | 3.9 | 1.8 | 1.9 | 2.3 | *i^N^*/*i* |
| DLY-12 | Roan | *GG* | 1.9 | 3.4 | 3.4 | 4.0 | 1.8 | 1.8 | 2.1 | *i^N^*/*i* |
| LL-1 | White | *AG* | 3.6 | 5.1 | 4.3 | 12.5 | 1.4 | 1.2 | 3.5 | *I^N^*/*_* |
| LL-2 | White | *AG* | 6.4 | 5.8 | 6.4 | 15.4 | 0.9 | 1.0 | 2.4 | *I^N^*/*_* |
| LL-3 | White | *AG* | 3.2 | 7.0 | 3.3 | 13.3 | 2.2 | 1.0 | 4.1 | *I^N^*/*i* |
| LL-4 | White | *AG* | 3.7 | 4.0 | 3.8 | 11.4 | 1.1 | 1.0 | 3.1 | *I^N^*/*_* |
| LL-5 | White | *AG* | 3.6 | 5.8 | 3.8 | 11.4 | 1.6 | 1.1 | 3.2 | *I^N^*/*_* |
| LL-6 | White | *AG* | 3.5 | 5.2 | 4.0 | 12.3 | 1.5 | 1.1 | 3.5 | *I^N^*/*_* |
| LL-7 | White | *AG* | 4.5 | 5.3 | 5.2 | 14.0 | 1.2 | 1.2 | 3.1 | *I^N^*/*_* |
| LL-8 | White | *AG* | 4.7 | 10.1 | 10.0 | 13.2 | 2.2 | 2.1 | 2.8 | *I*/*_* |
| LL-9 | White | *AG* | 5.1 | 12.5 | 10.0 | 14.5 | 2.5 | 2.0 | 2.8 | *I*/*_* |
| LL-10 | White | *AG* | 4.1 | 8.1 | 6.3 | 15.1 | 2.0 | 1.5 | 3.7 | *I*/*_* |
| YY-1 | White | *AG* | 2.8 | 7.4 | 3.0 | 9.1 | 2.6 | 1.1 | 3.3 | *I^N^*/*i* |
| YY-2 | White | *AG* | 3.6 | 6.2 | 3.9 | 11.4 | 1.7 | 1.1 | 3.2 | *I^N^*/*_* |
| YY-3 | White | *AG* | 3.2 | 7.6 | 5.4 | 10.7 | 2.4 | 1.7 | 3.3 | *I*/*i* |
| YY-4 | White | *AG* | 6.2 | 11.7 | 11.5 | 16.6 | 1.8 | 1.8 | 2.7 | *I*/*_* |
| YY-5 | White | *AG* | 4.4 | 9.2 | 8.8 | 15.3 | 2.1 | 2.0 | 3.5 | *I*/*_* |

DLY, Duroc × (Landrace × Large White); LL, Landrace; YY, Large White.

**Table S5** Copy number variations in the 561-kb region containing the *KIT* gene in 13 re-sequenced individuals

| Sample | Genomic copy number |
| --- | --- |
| DLY-1 | 4 |
| DLY-2 | 4 |
| DLY-3 | 3 |
| DLY-4 | 3 |
| DLY-5 | 2 |
| DLY-6 | 2 |
| LL-1 | 4 |
| LL-2 | 6 |
| LL-3 | 3 |
| LL-4 | 4 |
| YY-1 | 3 |
| YY-2 | 4 |
| YY-3 | 3 |

DLY, Duroc × (Landrace × Large White); LL, Landrace; YY, Large White.

**Table S6** Association of *KIT* genotypes and alleles with coat color phenotypes

| *KIT* genotype | Splice mutation | DUP2/DUP1 | DUP3/DUP1 | DUP4/DUP1 | Phenotype |
| --- | --- | --- | --- | --- | --- |
| *I(I^1^,I^2^ or I^3^)*/*i* | *AG* | 1.7-2.0  (n = 5) | 1.7-2.0  (n = 5) | 2.0-2.3  (n = 5) | White |
| *I^N1^*/*i or I^N1*^*/*i* | *AG* | 1.5-2.0  (n = 5) | 1.0  (n = 5) | 1.5-2.0  (n = 5) | White |
| *I^N1^*/*i or I^N1*^*/*i* | *AG* | 1.3  (n = 2) | 1.0  (n = 2) | 1.7-2.7  (n = 2) | White with reddish brown spots |
| *I^N1^*/*i or I^N1*^*/*i* | *AG* | 1.3  (n = 7) | 1.0  (n = 7) | 1.5-2.0  (n = 7) | Reddish brown with white legs and belts |
| *I^N2^*/*i or I^N2*^*/*i* | *AG* | 1.0  (n = 3) | 1.0  (n = 3) | 1.7-2.0  (n = 3) | White with reddish brown spots |
| *I^N2^*/*i or I^N2*^*/*i* | *AG* | 1.0  (n = 9) | 1.0  (n = 9) | 1.5-2.0  (n = 9) | Reddish brown with white legs and belts |
| *I^L^*/*i* | *AG* | 2.5  (n = 3) | 1.0  (n = 3) | 2.5  (n = 3) | White with reddish brown spots |
| *i^N1^*/*i* | *GG* | 2.0  (n = 4) | 2.0  (n =4) | 2.0  (n =4) | Roan |
| *i^N2^*/*i* | *GG* | 2.0  (n = 5) | 1.0  (n = 5) | 2.0  (n = 5) | Reddish brown with white belts |

Genomic copy numbers of DUP1-4 of these individuals were measured by ddPCR.

**Table S7** *KIT* genotypes and coat color phenotypes in 23 Duroc × Landrace hybrid piglets

| *KIT* genotype | Phenotype | No. | Splice mutation | DUP2/DUP1 | DUP3/DUP1 | DUP4/DUP1 |
| --- | --- | --- | --- | --- | --- | --- |
| *I^L^* /*i* | White with reddish brown spots | 4 | *AG* | 1.5-2.0 | 1.0 | 1.5-2.5 |
| *I^N2^*/*i* | Reddish brown with white spots | 19 | *AG* | 1.3 | 1.0 | 1.3 |
